# Dual Cancelled Channel STAP for Target Detection and DOA Estimation in Passive Radar

**DOI:** 10.3390/s21134569

**Published:** 2021-07-03

**Authors:** Giovanni Paolo Blasone, Fabiola Colone, Pierfrancesco Lombardo, Philipp Wojaczek, Diego Cristallini

**Affiliations:** 1 Department of Information Engineering, Electronics and Telecommunications (DIET), Sapienza University of Rome, 00184 Rome, Italy; fabiola.colone@uniroma1.it (F.C.); pierfrancesco.lombardo@uniroma1.it (P.L.); 2 Fraunhofer Institute for High Frequency Physics and Radar Techniques (FHR), 53343 Wachtberg, Germany; philipp.wojaczek@fhr.fraunhofer.de (P.W.); diego.cristallini@fhr.fraunhofer.de (D.C.)

**Keywords:** passive radar, GMTI, AB-STAP, DOA estimation

## Abstract

This paper deals with the problem of detection and direction of arrival (DOA) estimation of slowly moving targets against clutter in multichannel mobile passive radar. A dual cancelled channel space-time adaptive processing (STAP) scheme is proposed, aiming at reducing the system computational complexity, as well as the amount of required training data, compared to a conventional full array solution. The proposed scheme is shown to yield comparable target detection capability and DOA estimation accuracy with respect to the corresponding full array solution, despite the lower computational cost required. Moreover, it offers increased robustness against adaptivity losses, operating effectively even in the presence of a limited set of training data, as often available in the highly non-homogeneous clutter scenarios experienced in bistatic passive radar. The effectiveness of the proposed scheme and its suitability for passive GMTI are demonstrated against both simulated and experimental data collected by a DVB-T-based multichannel mobile passive radar.

## 1. Introduction

Recent advances in passive radar research have opened interesting new perspectives and application areas [1,2,3]. Among them, in the last few years, the potentialities of passive radar mounted onboard moving platforms have been investigated, aiming towards synthetic aperture radar (SAR) imaging [4,5,6,7] and ground moving target indication (GMTI) [8,9,10,11,12,13,14,15,16,17,18] applications.

One of the main obstacles to the use of passive radar on moving platforms is the motion-induced Doppler distortion of the received signals. On the one hand, this can complicate the reception and reconstruction of the reference signal [19,20]. On the other hand, in surveillance applications, the detection and direction of arrival (DOA) estimation of slowly moving target echoes are hindered by the Doppler-spread clutter returns from the stationary scene. This issue is exacerbated by the typical broad antenna beams available at VHF/UHF bands of the most widely used illuminators of opportunity. Therefore, a proper suppression of clutter signal is required. This can be achieved by operating in the space-time domain, exploiting systems featuring multiple channels on receive. However, numerous critical aspects brought in by the passive radar framework must be tackled, while possibly preserving the paradigm of a low-complexity system.

This topic has been recently addressed by these and other authors, with reference to passive radar sensors equipped with two or more channels on receive, and different effective solutions have been reported in the technical literature.

In [8,9,10,11,12,13], an approach based on displaced phase centre antenna (DPCA) was shown to be a particularly attractive and undemanding solution for passive radar applications in the case of dual-channel systems. However, the need to mitigate the potential angle-dependent channel imbalances requires the implementation of adaptive digital calibration strategies, operating across multiple processing stages, for a convenient suppression of direct signal interference and clutter [11,12,13]. Moreover, with two channels on receive, only the detection of target echoes can be sought, whereas the problem of its angular localisation within the broad antenna beam remains unsolved.

Such limitations can be overcome by employing multiple channels on receive and applying adaptive space-time processing (STAP) techniques [14,15,16,17,18]. Specifically, a complete scheme for STAP in mobile passive radar has been recently proposed by the authors in [18]. By resorting to a post-Doppler strategy [21,22,23], it easily fits into the typical passive radar processing chain and takes advantage of the long integration times to reduce the size of the adaptive cancellation problem and compensate for potential angle-dependent channel errors. A space-time detection scheme is then employed where a clutter-based calibration of the spatial steering vector allows for prevention of accidental rejection of the target echoes arising from a severe channel imbalance. Finally, the accurate target angular localisation is provided by a space-time maximum likelihood estimator (MLE).

The approach in [18] enables effective GMTI capability and gains a larger flexibility compared to the DPCA schemes thanks to the adaptive use of a higher number of spatial degrees of freedom (DOF). In fact, clutter cancellation, target detection and angular localisation, and even the spatial steering vector calibration are performed according to a fullarray strategy, i.e., by jointly exploiting all the *N* available channels on receive. This is paid in terms of an increased complexity for the resulting system, since it requires (i) the estimation and inversion of a (NL×NL) space-time disturbance covariance matrix, with *L* being the number of temporal DOF; (ii) the availability of an amount of training data greater than 2NL in order to limit the adaptivity loss, which might be difficult to be guaranteed in the considered bistatic passive radar scenario; and (iii) the implementation of computationally expensive algorithms for the maximisation of the DOA MLE likelihood function, with computational load being dependant on the desired estimation accuracy.

In this paper, we address the above limitations and propose an alternative approach based on a dual cancelled channel STAP scheme. This approach takes inspiration from a family of sub-optimal STAP schemes reported in the active radar literature [24,25,26,27], where an adaptive transformation is first applied on the received data to reduce the number of spatial DOF, which are then directly exploited for target detection and DOA estimation. For instance, the generalised monopulse estimator (GME) in [25] relies on the sum and difference channels obtained after an adaptive clutter cancellation. However, in order to also limit the computational cost of the preliminary adaptive transformation, we resort to the AB-STAP technique presented by the authors in [26].

This technique is first integrated into the post-Doppler STAP scheme proposed in [18]. After the first processing stages are applied separately at each receiving channel, the outputs are arranged into two spatially displaced antenna sub-arrays, and a STAP scheme is applied to each of them to obtain two clutter cancelled channels. These channels are then adaptively recombined for the purpose of target detection, while target DOA can be estimated by exploiting their different phase centres. The AB-STAP approach further reduces the computational complexity of the system by lowering the number of the adaptive DOF in the space-time clutter filtering (namely, the size of the covariance matrices to be estimated and inverted). Despite the lower computational cost, the proposed scheme does not suffer from significant performance losses with respect to the conventional full-array solution. Moreover, it is more robust against adaptivity losses, operating effectively even in the presence of a limited sample support. This plays a key role in a real passive radar scenario, where the non-homogeneity of the clutter response may limit the number of relevant training data, affecting the STAP performance. Lastly, the target DOA estimation does not involve a functional maximisation, thanks to the closed form expression of the estimator, thus requiring a lower computational cost, which in turn is independent of the desired estimation accuracy.

The moving target detection and localisation performance of the proposed strategy were analysed and compared with those of the equivalent full array solution (assumed as a benchmark), first in a simulated scenario and then against experimental data collected by a DVB-T based multichannel passive radar mounted on a moving platform.

The rest of the paper is organised as follows. In Section 2, the processing scheme for passive radar STAP proposed in [18] is recalled. In Section 3, we describe the processing steps of a dual cancelled channel STAP scheme for passive radar. Its performance is then analysed in Section 4, both in terms of target detection and DOA estimation, against a simulated clutter scenario and compared with that of the corresponding full array solution. In Section 5, the effectiveness of the proposed scheme is validated against a set of experimental data. Finally, we draw our conclusions in Section 6.

## 2. Passive Radar STAP Scheme

In this section, we briefly recall the processing scheme proposed in [18] for passive radar STAP, here referred to as fullarray STAP since it jointly exploits all the available spatial DOF. The interested reader is referred to [18] for a more detailed description.

We assumed the same signal and clutter model adopted in [10], for a dual channel system, and in [18], for the corresponding multichannel case.

**Figure 1:**
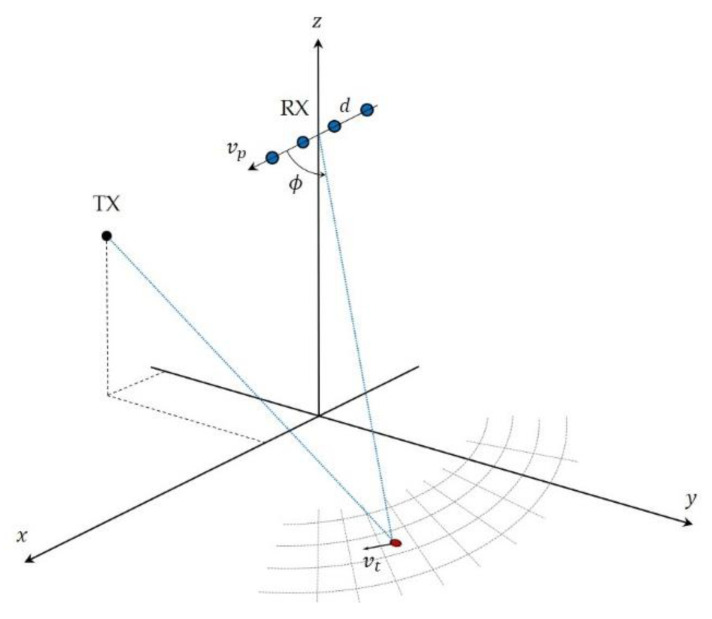
Considered system geometry for a multichannel mobile passive radar exploiting a stationary transmitter as illuminator of opportunity.

We considered a radar receiver installed on a platform moving at velocity $v_{p}$ and exploiting a stationary illuminator of opportunity (Figure 1). The N receiving channels, displaced by d in the along-track direction, form a side-looking uniform linear array (ULA). We denote by ϕ the angle between antenna end-fire and receiver to scatterer line of sight.

After a preliminary stage including synchronisation and reconstruction of the reference signal, a batch processing architecture is separately applied at each receiving channel, recreating the conventional fast-time/slow-time framework of a pulsed radar operating at an equivalent pulse repetition time (PRT) given by the batch duration T.

For each batch, the range compression is performed by resorting to a reciprocal filtering strategy [28,29,30,31]. This has the role of both improving the signal ambiguity function and providing a time-invariant impulse response by eliminating the temporal variability associated to the employed waveform of opportunity [10].

The range compressed data from the cell under test are arranged into a (NM×1) space-time vector $\tilde{x}$, with M being the number of batches in the coherent processing interval (CPI). In the target presence, this is expressed as $\tilde{x}=A\;\tilde{s}+\tilde{d}$, where A denotes the target complex amplitude; $\tilde{s}$ the space-time steering vector; and $\tilde{d}$ a disturbance vector, assumed Gaussian with covariance matrix $\tilde{Q}$. For a side-looking ULA, vector $\tilde{s}\left(f_{D},\phi \right)=s_{t}\left(f_{D}\right)\;⨂\;s_{s}\left(\phi \right)$ has time and space components $s_{t}\left(f_{D}\right)={\left[1,e^{-j2\pi f_{D}T},\ldots ,e^{-j2\pi f_{D}MT}\right]}^{H}$ and $s_{s}\left(\phi \right)={\left[1,e^{j2\pi d/\lambda \cos\phi },\ldots ,e^{j2\pi Nd/\lambda \cos\phi }\right]}^{H}$, $f_{D}$ denoting the target Doppler frequency, λ the carrier wavelength, H the Hermitian transpose, and ⨂ the Kronecker product.

In [18], a post-Doppler STAP approach [21,22,23] is suggested as appropriate for mobile passive radar, where the typical long integration times provide a good decoupling of the clutter Doppler components. The same adjacent-bin post-Doppler (ABPD) strategy is adopted here by operating adaptively on the N spatial channels and a subset of L adjacent Doppler processed data around the cell under test.

We denote by x=A s+d the (NL×1) data vector in the post-Doppler domain, where $s\left(\phi \right)=s_{d}⨂s_{s}\left(\phi \right)$ is the steering vector and Q the disturbance covariance matrix. The well-known adaptive matched filter (AMF) detector [32] is given by

(1) $$\frac{{\left|s^{H}{\hat{Q}}^{-1}x\right|}^{2}}{s^{H}{\hat{Q}}^{-1}s\;}≷\eta$$

where η is the detection threshold, selected according to a desired false alarm probability (PFA). Matrix Q is replaced with its ML estimate $\hat{Q}=X_{k}X_{k}^{H}$, obtained by a set of training data $X_{k}=\left[x_{1},\ldots ,x_{K}\right]$ from K adjacent range cells, assumed independent, identically distributed and target-free.

Once a target is detected, an asymptotically efficient estimate of its DOA is given by the MLE [33], which takes the maximum of the log-likelihood function with respect to ϕ:

(2) $${\hat{\phi }}_{t}=\underset{\phi }{\arg\;\max}\left\{\frac{{\left|s^{H}\left(\phi \right){\hat{Q}}^{-1}x\right|}^{2}}{s^{H}\left(\phi \right){\hat{Q}}^{-1}s\left(\phi \right)\;}\right\}.$$

The above STAP scheme proved effective for passive radar GMTI, but it involves a non-negligible level of complexity. It requires the estimation and inversion of a (NL×NL) disturbance covariance matrix, potentially for each range-Doppler bin. Moreover, target DOA estimation requires finding the maximum of a non-linear function, either by fast converging algorithms or by evaluating it for a set of values and selecting the one yielding the maximum. This might be computationally expensive, the cost being proportional to the desired estimator accuracy. Such cost may not be in line with the usual low complexity of passive radar, especially in the case of mobile systems offering onboard processing.

In addition, the availability of a sufficient number of uniform training data to limit the adaptivity loss may be difficult to be guaranteed in a passive bistatic radar scenario. In fact, the typical ground-based transmitters of opportunity are likely to produce a non-uniform illumination of the ground due to the masking effects of terrain. Moreover, due to its silent nature and potentially limited coverage, passive radar can be operated also at short range where non-uniform clutter distributions occur, especially for airborne acquisition geometry.

To mitigate the above limitations, we propose an alternative STAP approach for target detection and DOA estimation in an operational mobile passive radar.

## 3. Dual Cancelled Channel STAP for Passive Radar

The dual cancelled channel STAP approach adaptively forms two clutter cancelled channels that are then directly exploited for both target detection and DOA estimation. Specifically, we consider the AB-STAP technique, originally presented in [26]. This is particularly suitable for the passive radar case, thanks to its simple architecture, reduced computational load, and robustness against highly non-homogeneous clutter scenarios.

**Figure 2:**
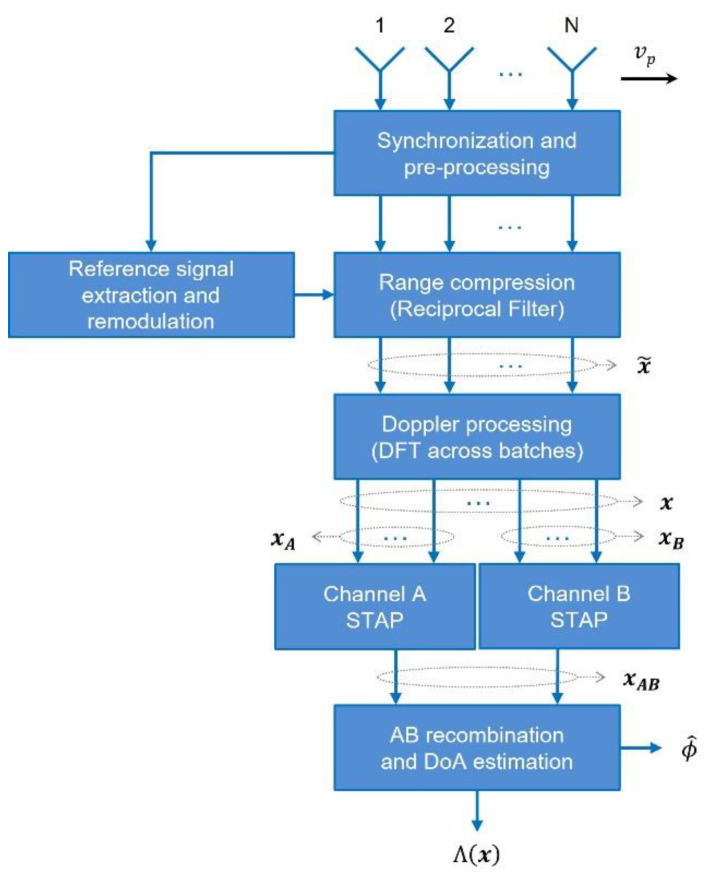
Processing scheme for post-Doppler AB-STAP in passive radar.

The adopted scheme is sketched in Figure 2. It is obtained by modifying the scheme proposed in [18], so as to integrate the considered dual cancelled channel approach. Specifically, the processing steps that characterise the AB-STAP scheme, and that differentiate it from the original one, develop downstream of the post-Doppler transformation stage.

These processing steps are described in the following:

(i) Formation of two spatially displaced channels: dividing the antenna array into two (possibly overlapped) sub-arrays, the data vector x is split into two $\left(N_{0}L\times 1\right)$ vectors, $x_{A}$ and $x_{B}$, corresponding to channels A and B, respectively. One includes the samples from the first $N_{0}$ antennas, the other the samples from the last $N_{0}$ antennas $\left(N/2\leq N_{0}<N\right)$.

(ii) Cancellation of clutter on each channel: the same post-Doppler STAP is applied on both channels by filtering $x_{A}$ and $x_{B}$ with a sample matrix inverse scheme and obtaining

(3) $$y_{A\left(B\right)}=w_{A\left(B\right)}^{H}x_{A\left(B\right)}=s_{A\left(B\right)}^{H}\left(\phi_{L}\right){\hat{Q}}_{A\left(B\right)}^{-1}x_{A\left(B\right)}$$

where $s_{A\left(B\right)}\left(\phi_{L}\right)$ and ${\hat{Q}}_{A\left(B\right)}$ are the steering vector and the estimated covariance matrix of channel A (B), respectively. Notice that, while ϕ represents the generic unknown target DOA, we denote by $\phi_{L}$ the array look direction.

This contributes to the reduction in the computational complexity, since it involves a smaller size of the inverse matrix operation. Moreover, in a uniform linear array, channels A and B exhibit disturbance with the same structure, being $s_{B}\left(\phi_{L}\right)=s_{A}\left(\phi_{L}\right)\exp\left\{j2\pi \left(N-N_{0}\right)d/\lambda \cos\phi_{L}\right\}$. As a result, a better estimate can be obtained by averaging the estimates made on the two channels, that is, ${\hat{Q}}_{A}={\hat{Q}}_{B}=\frac{1}{2}\left(X_{kA}X_{kA}^{H}+X_{kB}X_{kB}^{H}\right)$, with $X_{kA}$ and $X_{kB}$ being the $\left(N_{0}L\times K\right)$ sets of training data for channels A and B, respectively. Although not totally independent (if $N_{0}>N/2$), averaging the two estimates yields a more stable estimate of the true covariance matrix, as well as a single matrix inversion.

(iii) Recombination of channels for target detection: using only one of the two channels to detect targets may result in significant performance degradation in terms of cancellation capability, with the equivalent antenna length and employed spatial DOF being reduced. To recover this degradation and avoid detection losses, an optimal coherent recombination of the two channels is performed by solving a small (2×2) adaptive problem.

Arranging the two outputs in the (2×1) vector $x_{AB}={\left[y_{A}\;y_{B}\right]}^{T}$ and applying the AMF detection scheme, we obtain

(4) $$\frac{{\left|s_{AB}^{H}{\hat{Q}}_{AB}^{-1}x_{AB}\right|}^{2}}{s_{AB}^{H}{\hat{Q}}_{AB}^{-1}s_{AB}\;}≷\eta_{AB}$$

where $\eta_{AB}$ is the detection threshold, and the steering vector $s_{AB}$ is given by

(5) $$s_{AB}\left(\phi_{L}\right)=\left[\begin{array}{c} w_{A}^{H}s_{A}\left(\phi_{L}\right)\\ w_{B}^{H}s_{B}\left(\phi_{L}\right) \end{array}\right]=w_{A}^{H}s_{A}\left(\phi_{L}\right)\left[\begin{array}{c} 1\\ 1 \end{array}\right].$$

${\hat{Q}}_{AB}$ is the (2×2) estimated covariance matrix at the output of channels A and B. Notice that $Q_{AB}$ is a Toeplitz Hermitian matrix with terms α and ρ respectively being the average disturbance power at the output of the two channels and their cross-correlation. This aspect can also be exploited to obtain a more stable estimate from the data.

The optimal recombination of the two channels allows for recovery of most of the detection loss with respect to the full array case [26]. Moreover, due to the smaller number of adaptive DOF used by each step of the AB-STAP approach, a lower computational cost as well as reduced adaptivity losses are expected in comparison to full array STAP. This latter aspect in particular plays a fundamental role in a real passive radar scenario, where the potential non-homogeneity of bistatic clutter may limit the number of relevant training data usefully exploitable for filter adaptivity.

(iv) Estimation of target DOA from the two cancelled channels: for range cells where a detection is declared, target DOA can be estimated from the two cancelled channels exploiting their different phase centres. Applying the MLE to the two outputs results in

(6) $${\hat{\phi }}_{t,AB}=\underset{\phi }{\arg\;\max}\left\{\frac{{\left|s_{AB}^{H}\left(\phi \right){\hat{Q}}_{AB}^{-1}x_{AB}\right|}^{2}}{s_{AB}^{H}\left(\phi \right){\hat{Q}}_{AB}^{-1}s_{AB}\left(\phi \right)\;}\right\}.$$

The maximisation is performed only with respect to the last stage of the processing chain, which only involves two-dimensional quantities. This allows us to find a closed form expression for the ML estimate of target DOA.

By defining the quantities $z={\left[\begin{array}{cc} z_{1} & z_{2} \end{array}\right]}^{T}=Q_{AB}^{-1}x_{AB}$, $v=z_{1}z_{2}^{H}/{\left|z\right|}^{2}$, and u=ρ/α, we obtain the ML estimate of target DOA as (see [27] for more details)

(7) ψ^=arcsin(2Im{vuH}|2v+u|)−∠(2v+u)

(8) $${\hat{\phi }}_{t,AB}=\arccos\left\{\frac{\lambda }{2\pi d\left(N-N_{0}\right)}\hat{\psi }+\cos\phi_{L}\right\}.$$

where Im{ζ} and ∠(ζ) are imaginary part and phase of complex scalar ζ, respectively.

The obtained DOA estimator does not involve a functional maximisation and it is implemented by a simple closed form expression, thus limiting the computational cost.

It is worth noting that the value of $N_{0}$ defines the number of spatial DOF used for clutter rejection on channels A and B, but it also affects the distance of their phase centres. Therefore, a trade-off exists between the theoretical sensitivity of DOA estimation and the signal-to-clutter plus noise ratio (SCNR) achievable at the output of the channels. Appropriate choices should be made according to the specific scenario. Notice that the SCNR at the output of the A and B channels, responsible for DOA estimation accuracy, is always lower than the SCNR available for target detection after channel recombination.

## 4. Performance Analysis in Simulated Scenarios

In this section, we analyse the effectiveness of the AB-STAP approach for a multichannel mobile passive radar, and we compare its performance with respect to the corresponding full array solution in terms of target detection and DOA estimation capability. To this purpose, we tested the considered schemes against a simulated clutter scenario.

We considered the case of a side-looking airborne receiver exploiting a DVB-T transmitter as illuminator of opportunity. Starting from a simulated reference signal, the clutter returns were generated, assuming a distribution of stationary and independent scatterers according to the model in [10]. Table 1 summarises the main simulation parameters. 

A clutter-to-noise ratio (CNR) of 20 dB was set at the input of each channel before range compression. Two identical moving targets were simulated at bistatic range $R_{b}=2\;\mathrm{km}$ and 5 km, having DOA $\phi_{t}=90{}^{\circ}$, bistatic radial velocity $v_{b}=8\;m/s$ ($f_{D}≅18\;\mathrm{Hz}$), and signal-to-noise power ratio (SNR) −50 dB at the input of each channel.

**Figure 3:**
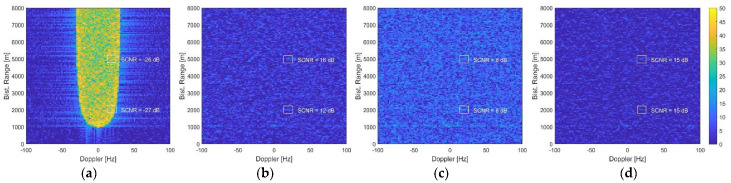
Range-Doppler maps from the simulated clutter scenario: (**a**) single channel; (**b**) after full array STAP with K = 24 training data; (**c**) after full array STAP with K = 16 training data; (**d**) after AB-STAP with K = 16 training data.

In Figure 3a, the range-Doppler map obtained from a single channel is reported. As is evident, the non-negligible receiver altitude caused a non-uniform distribution of clutter in the near range region, where the first target was located. In contrast, in the far range region of the second target, clutter distribution was uniform, spreading over a Doppler bandwidth of approximately $2v_{p}/\lambda ≅60\;Hz$. The resulting target SCNR after range compression and Doppler processing was −27 dB and −26 dB, respectively, so that both targets were completely buried into clutter returns. The SCNR was measured as the ratio between the power level at the target range-Doppler location, when the processing was fed only with the target echo, and the disturbance power level estimated on the area surrounding target location, when only clutter and noise were present.

Figure 3b shows the range-Doppler map obtained when applying the full array STAP scheme, using N=4 channels, L=3 adjacent Doppler bins, and K=2NL=24 training data. The clutter was effectively cancelled, and the SCNR of the target at far range was brought to 16 dB, with an improvement of 42 dB. For the near range target instead, the achievable SCNR was limited to 12 dB, due to the inhomogeneity of clutter data. To avoid this, in Figure 3c, the sample support was reduced to K=16. In this case, however, the small number of training data yielded higher adaptivity losses, rising the residual disturbance background and limiting the final SCNR to 8 dB for both targets.

The result obtained when applying the AB-STAP scheme with K=16 training data is shown in Figure 3d. The array was split into two non-overlapped sub-arrays of $N_{0}=2$ antennas, forming the A and B channels. With N=4 elements at disposal, this choice ensured the largest phase centre displacement. In this case, the clutter was still effectively suppressed, and the limited number of secondary data counteracted the effect of non-homogeneous clutter at near range, without introducing undesirable adaptivity losses. As a result, a final SCNR of 15 dB was obtained for both the far and the near range targets.

### 4.1. Target Detection Performance

To compare the fullarray and the AB-STAP schemes in terms of detection performance, we carried out a Monte Carlo analysis under different conditions of availability of the secondary data. We assumed the same parameters used in the previous example and, for simplicity, a uniform clutter scenario. The space-time AMF detectors in (1) and (4) were considered, with desired PFA level 10^−4^. The Swerling 0 target model was assumed.

**Figure 4:**
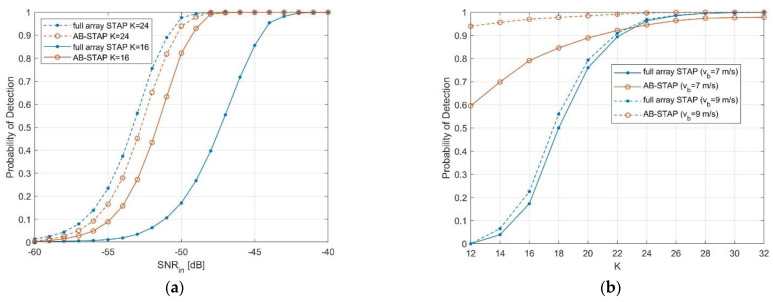
Detection performance comparison: (**a**) as a function of target input SNR, for target bistatic velocities 7 m/s; (**b**) as a function of the number of training data, for target input SNR −50 dB. The desired PFA was set to 10^−4^.

In Figure 4a, the resulting probability of detection (PD) is shown as a function of the target input SNR. The target bistatic radial velocity $v_{b}=7\;m/s$ was representative of a slow target condition, sufficiently close to the clutter notch. Two cases were considered, with different amounts of available training data. Specifically, K=24 represents the case of enough training data for the full array solution (twice the number of the adaptive DOF), while K=16 represents a condition of a limited sample support.

When K=24 training data are available (dashed curves), AB-STAP performs very closely to full array STAP. Although clutter cancellation is performed by means of only two spatial DOF on the A and B channels, their optimal coherent recombination allows us to avoid considerable detection losses. Notice that the slight remaining advantage of the full array case (<1 dB) can be further reduced if considering target velocities further from clutter notch or by adopting overlapped solutions for A and B channels (e.g., $N_{0}=3$).

When the amount of training data decreased to K=16 (solid curves), significant performance degradation was observed in the full array case, due to adaptivity losses. Conversely, the AB-STAP only showed a limited performance drop, thanks to the smaller number of adaptive DOF used in each step (half of the full array case). As a result, in this case, AB-STAP outperformed full array STAP by approximately 5 dB.

The above considerations find confirmation also in Figure 4b, where the PD was evaluated as a function of K, for a fixed target SNR of −50 dB. As expected, the performance decreased significantly more in the full array case, as the number of training data reduced, while losses associated with the AB-STAP approach were remarkably smaller. Moreover, even for larger values of K, AB-STAP showed minor differences in performance, compared to full array STAP, only for very slow targets, close to clutter notch. Moreover, this was obtained in favour of a lower complexity and computational cost.

### 4.2. DOA Estimation Performance

As discussed in Section 3, once a target is detected, AB-STAP is able to perform the target DOA estimation via the closed form expression in (7) and (8) by exploiting the outputs of the A and B channels. The DOA estimation accuracy provided by this approach was evaluated and compared with the full array STAP estimator in (2) by a Monte Carlo analysis applied against the same simulated scenario. Specifically, the full array MLE was operated with a bank of filters equally spaced in angle by δϕ=0.1° within the array nominal beamwidth (BW ≅38°), corresponding to a step smaller than BW/100. The AB-STAP scheme employed $N_{0}=2$ elements for the A and B channels. Both the estimators were applied after the post-Doppler transformation with L=3. The bistatic velocity and DOA of the simulated target were respectively $v_{b}=7\;m/s$ and $\phi_{t}=\phi_{L}=90{}^{\circ}$.

**Figure 5:**
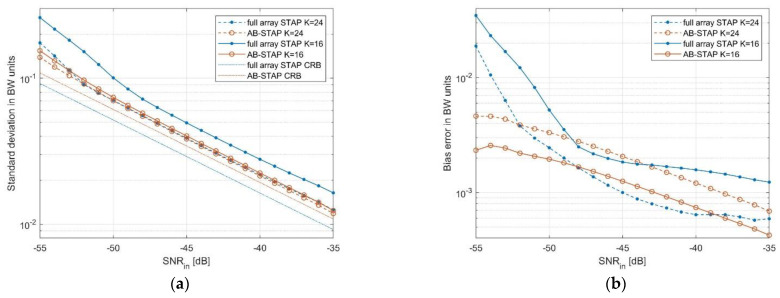
Comparison of DOA estimation accuracy as a function of the target input SNR: (**a**) standard deviation in BW units; (**b**) bias error in BW units.

In Figure 5, the resulting estimation accuracy is shown as a function of target input SNR. The results are compared in terms of standard deviation in Figure 5a and bias error in Figure 5b, both normalised to the nominal BW.

We notice that, for K=24 training data (dashed curves), the accuracy of AB-STAP and full array STAP estimators were comparable, showing an almost identical standard deviation. When the training data was lowered to K=16 (solid curves), the standard deviation and bias error increased in the full array case. Instead, the AB-STAP showed negligible adaptivity losses even for smaller sample support. In this case, AB-STAP allowed for an improvement of the standard deviation by approximately 20% with respect to full array STAP.

**Figure 6:**
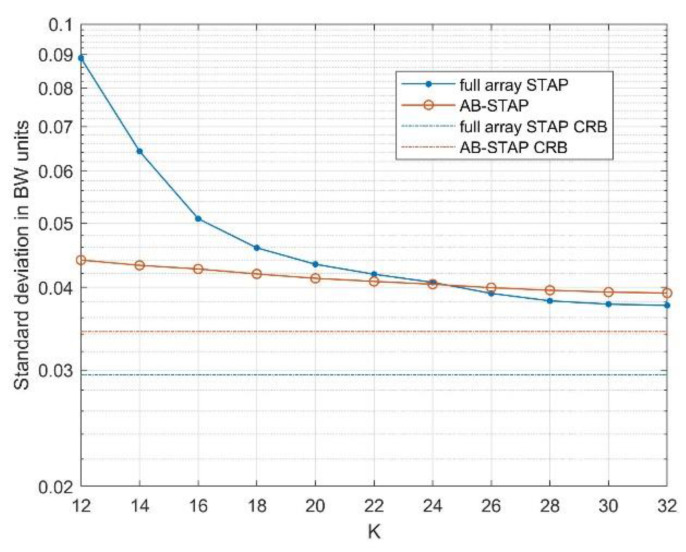
Comparison of DOA estimation accuracy as a function of the amount of training data. Target input SNR was set to −45 dB and bistatic velocity to 7 m/s.

The above considerations are also supported by the results in Figure 6, where the DOA estimation performance is represented as a function of K, for a fixed SNR of −45 dB.

In Figure 5a and Figure 6, the Cramér–Rao bound (CRB) is also reported for both the considered estimators (dash-dotted curves). This provides a useful reference for the DOA estimation accuracy and allows for better assessment of the effect of the selected strategies on the ideal estimation performance.

From [34,35], the CRB for the estimator in (2) can be expressed as

(9) $$\sigma_{\phi }^{2}={\left\{2{\left|A\right|}^{2}\left[\left({\dot{s}}^{H}\left(\phi \right)Q^{-1}\dot{s}\left(\phi \right)\right)-\frac{{\left|{\dot{s}}^{H}\left(\phi \right)Q^{-1}s\left(\phi \right)\right|}^{2}}{s^{H}\left(\phi \right)Q^{-1}s\left(\phi \right)}\right]\right\}}^{-1}$$

where $\sigma_{\phi }$ is the standard deviation of the estimation error and $\dot{s}\left(\phi \right)=\partial s\left(\phi \right)/\partial \phi$. The corresponding CRB for the AB-STAP scheme can be obtained by replacing s, $\dot{s}$, and Q with their corresponding two-dimensional quantities, $s_{AB}$, ${\dot{s}}_{AB}$, and $Q_{AB}$.

Both the estimators appeared as asymptotically unbiased and efficient due to their ML nature. The amount of loss of the simulated results with respect to the CRB is a measure of the adaptivity loss, due to the available training data. Again, this confirms the robustness of the AB-STAP approach. In fact, despite its slightly worse asymptotic performance (see the difference in the CRB curves), it performed better than the full array scheme, as the availability of training data reduced.

Finally, it is worth recalling that the full array estimator requires finding the maximum among a discrete set of angles (bank of filters) for DOA estimation. Therefore, it can be subject to performance saturation effects and involves a non-negligible computational load, which increases with the desired accuracy. Conversely, the closed form estimator of the AB-STAP scheme is not affected by saturation and offers a reduced complexity and computationally attractive solution.

## 5. Experimental Results

**Figure 7:**
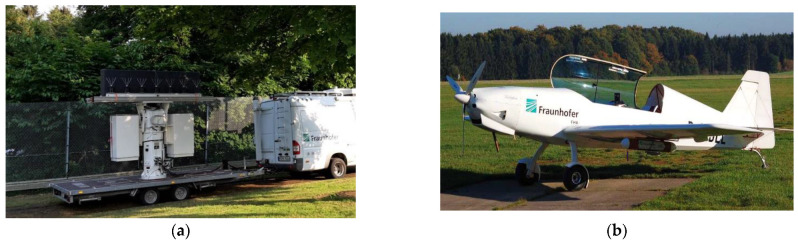
(**a**) The experimental radar system installed behind a vehicle in side-looking. (**b**) The ultralight aircraft (Delphin).

This section provides an experimental validation of the effectiveness of the AB-STAP scheme for moving target detection and localisation in mobile passive radar. The data are acquired by a multichannel receiver developed by Fraunhofer FHR, mounted on a ground moving vehicle and exploiting DVB-T transmissions (see Figure 7a). The experimental setup and the considered dataset were formerly used in [18], where the full array STAP scheme was successfully applied. In this paper, the full array solution is compared with the AB-STAP approach in order to verify the benefits of the latter in a real scenario.

The campaign was carried out in a rural area in western Germany. The selected illuminator of opportunity was the Eifel DVB-T transmitter. The receiver configuration consisted of two PARASOL units [36], each providing two receiving channels. The four available channels were displaced in the along-track direction to form a side-looking ULA. An absorbing material was used to attenuate possible back lobe returns. The available channels were all employed as surveillance channels, while the reference signal was reconstructed by resorting to a decode/recode strategy.

**Figure 8:**
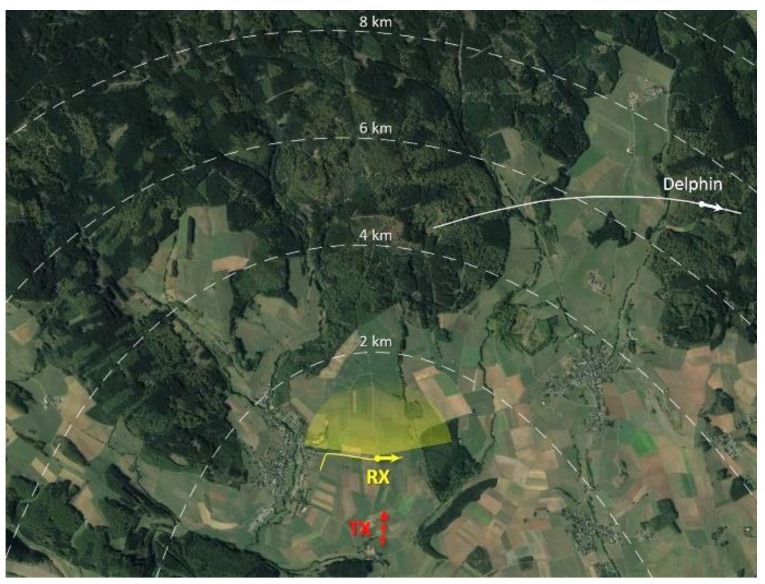
Optical image of the acquisition area. The position and direction of motion of the receiver and the aerial target are indicated with yellow and white arrows, respectively. Red arrow indicates the transmitter direction of arrival. Dashed lines represent the bistatic iso-range curves.

Figure 8 shows a sketch of the bistatic acquisition geometry. The transmitter was in a direction approximately opposite to the observed scene. An ultralight aircraft (Delphin) was employed as a cooperative target during the acquisition campaign (see Figure 7b). In addition to the real target, four simulated moving targets were injected into the acquired data. The parameters of the real and simulated targets are reported in Table 2.

It is worth noting that the same strategy presented in [18] for calibration of the spatial steering vector component was adopted here in order to mitigate the impact of the potential angle-dependent inter-channel imbalance on the target detection and localisation performance. Specifically, this was applied to vector s in the full array STAP scheme and equivalently to vectors $s_{A}$ and $s_{B}$ in the AB-STAP scheme.

**Figure 9:**
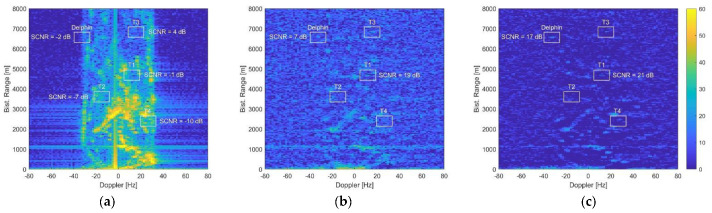
Range-Doppler maps resulting from the experimental data: (**a**) single channel map; (**b**) after full array STAP; (**c**) after AB-STAP. The amount of training data was set to K = 16. The spatial steering vector was towards the direction of targets T1 and Delphin. Target positions are indicated by white boxes. SCNR is reported after STAP only for targets included in the resulting main beam.

Figure 9 reports the range-Doppler maps resulting from a CPI of ≈ 0.57 s (corresponding to 512 OFDM symbols of the DVB-T signal). In particular, Figure 9a shows the map resulting from a single channel, namely, before STAP, normalised to the noise power level. Given a platform velocity of 13.8 m/s and a carrier wavelength of 0.43 m, the stationary scene echoes appeared to spread over a Doppler bandwidth of approximately 64 Hz.

The radial velocities and DOAs of the real and simulated moving targets were such that they fell within Doppler bandwidth of clutter, being mostly buried into it and therefore not easily detectable. Notice the average SCRN values were higher compared with those of the simulated case, since targets compete with a lower local clutter power.

The observed clutter scenario was characterised by a large heterogeneity, associated with the presence of densely vegetated and rural areas. This aspect was exacerbated by the bistatic geometry of the passive radar exploiting a ground-based transmitter, prone to non-uniform illumination and shadowing phenomena, due to the orography of terrain. In this context, the AB-STAP approach represents a suitable solution, since it operates effectively even with a limited sample support.

Figure 9b shows the range-Doppler map obtained after applying the full array STAP scheme, which jointly uses the N=4 channels and L=3 Doppler bins. The amount of exploited training data was set to K=16. Notice that this amount was less than twice the number of adaptive DOF for the full array case (2NL=24). As expected, the large adaptivity losses resulted in a high residual disturbance, which lowered the achievable SCNR and may hinder the detection of targets.

For comparison, Figure 9c reports the corresponding range-Doppler map obtained after applying the AB-STAP scheme, using $N_{0}=2$ elements for the A and B channels and employing the same amount of training data.

**Figure 10:**
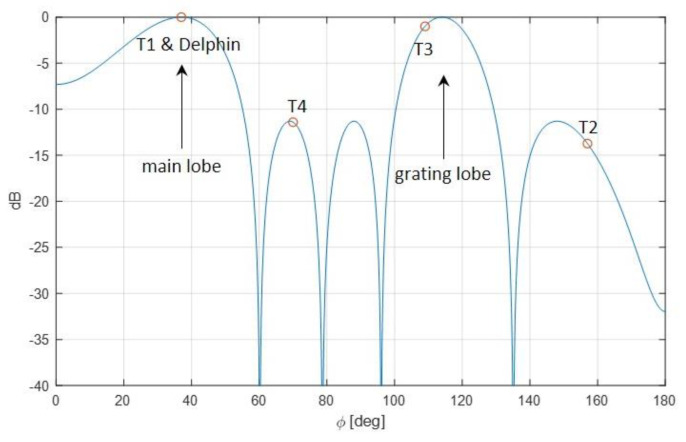
Theoretical array pattern for steering at ϕ = 37°. Target positions are indicated by red circles.

In both cases, the spatial steering vector was oriented in a direction ($\phi_{L}=37{}^{\circ}$) such as to include in the main beam the real target Delphin and target T1, which both appear clearly visible in the final maps. Therefore, the SCNR obtained after STAP was indicated in the figure only for these targets. The other targets, whose DOAs were not included in the main beam, are less visible. The theoretical array pattern is shown in Figure 10, with the corresponding target angular positions. Notice that, due to the element spacing d≅0.83λ, grating lobes arose for steering directions far from the broadside. In the considered case, target T3 is also visible in the map since located in direction of a grating lobe.

The above result clearly demonstrates the effective clutter suppression and moving target detection capability of the AB-STAP approach, also in the presence of a limited sample support for the adaptive filter estimation.

**Figure 11:**
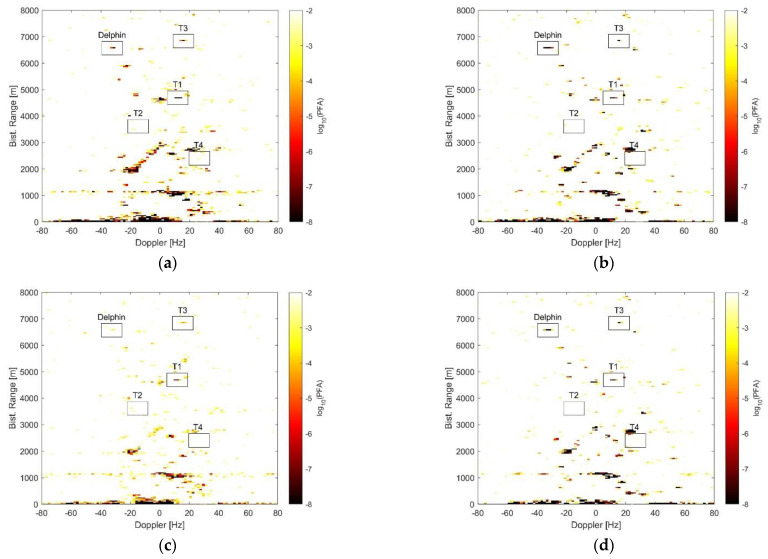
Minimum nominal PFA to detect each bin: (**a**) full array STAP with K = 24; (**b**) AB-STAP with K = 24; (**c**) full array STAP with K = 16; (**d**) AB-STAP with K = 16. Values are expressed as $\log_{10}\left(\mathrm{PFA}\right)$. Spatial steering vector is towards the direction of targets T1 and Delphin. Target positions are indicated by black boxes.

For a more comprehensive analysis and comparison of the detection performance, Figure 11 shows the results obtained with the full array STAP detector in (1) and with the AB-STAP detector in (4) by reporting their test statistics over the range-Doppler map, mapped into the nominal PFA values that would yield a detection. Basically, each range-Doppler bin was scaled to represent the minimum nominal PFA for that bin to be detected by the corresponding detection scheme.

Specifically, the two schemes are compared in Figure 11a,b for K=24 training data and, similarly, in Figure 11c,d for K=16. Also in this case, the spatial steering vector was oriented towards the DOA of Delphin and target T1.

For a full comparison, Table 3 reports the minimum PFA that allows for the detection of each target when an appropriate spatial steering includes the target in the main beam.

From the results in Figure 11 and Table 3, the following considerations are in order:

- Comparing Figure 11a,b, the full array STAP yielded a higher number of false alarms compared to the AB-STAP for a given PFA. The false alarms were mostly associated with persistent clutter structures, especially in those areas where the clutter power showed abrupt variations and the filter adaptivity was more likely to fail.
- It is worth mentioning that the presence in the observed scene of other non-cooperative moving targets during the acquisition cannot be excluded.
- When the training data were reduced to K=16 (Figure 11c,d), the more localised adaptation capability allowed the AB-STAP scheme to better handle the clutter discrete and reduce the number of false alarms without compromising target detection. Conversely, in the full array STAP scheme, this was paid in terms of higher adaptivity loss, which raised the detection threshold to prevent a general increase of the false alarms, thus compromising the detection capability.
- Looking at Table 3, when enough training data were available, both the detection schemes yielded remarkable results, allowing for target detection until low values of PFA. In the case of limited sample support, instead, the detection performance of full array STAP drastically decreased, while the AB-STAP approach was able to mostly preserve or even improve its outcomes (see for example T2 and T4). As a result, AB-STAP largely outperformed the full array STAP for all targets in this case.
- By selecting a PFA of $10^{-4}$, with K=24 training data, all the considered targets would be detected by both the detection schemes. For K=16, the AB-STAP would still detect all the targets, while the full array scheme would miss the detection of three out of five targets.

After target detection, a proper estimation of target DOA was worthwhile for target localisation purposes. In fact, the array nominal BW was in the order of 23° (at broadside), thus providing poor target localisation capability. This was achieved by resorting to the space-time ML DOA estimation strategies formerly presented.

In the full array STAP case, target angular position was estimated by finding the maximum of (2) over a bank of filters equally spaced by δϕ=0.1° within the nominal BW, corresponding to a step smaller than BW/100. In the AB-STAP case instead, the closed form expression in (8) was exploited. For the purpose of our analysis, we neglected the angular ambiguity resulting from the antenna spacing larger than λ/2, and we used the known target position in the estimation process to identify the non-ambiguous angular sector. Please note that this strategy did not affect our results, as we were mostly interested in small estimation errors around the true target DOA.

The results of DOA estimation for each target are reported in Table 4 for an amount of training data equal to K=24 and K=16. The error with respect to the true target DOA values in Table 2 is reported in brackets.

Analysing the results, we notice that for a large sample support, both the AB-STAP and the full array STAP provided an accurate DOA estimation for all targets, with an average error below one-twentieth of the nominal BW. When the sample support was limited to K=16 instead, the estimation accuracy of the full array STAP was considerably reduced. On the other hand, the AB-STAP approach was able to mostly preserve its good localisation performance, while requiring a lower computational complexity.

**Figure 12:**
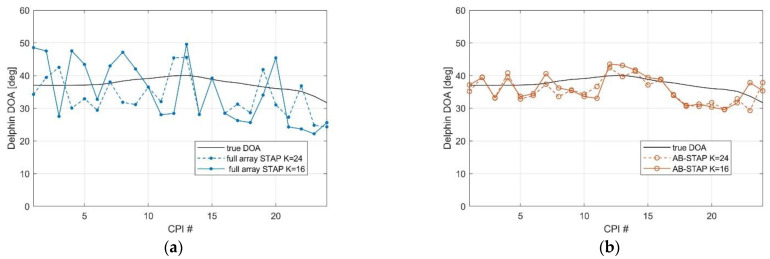
DOA estimation results for the aerial target Delphin over consecutive CPIs: (**a**) full array STAP; (**b**) AB-STAP.

Finally, Figure 12 shows the DOA estimation results obtained for the cooperative aerial target Delphin over consecutive scans. Specifically, 24 subsequent CPIs were considered, each of length 512 OFDM symbols and overlapped by 256 symbols, for an overall observation time of approximately 7 s. The results clearly showed the advantage of the AB-STAP scheme compared to the full array scheme, both in terms of accuracy and stability of the estimation, especially in the case of a limited sample support.

The results reported in this section prove the capabilities of the AB-STAP strategy against a real clutter scenario and its advantages over the full array scheme. The AB-STAP represents a suitable and convenient solution for target detection and DOA estimation in mobile passive radar equipped with multiple channels on receive.

## 6. Conclusions

In this paper, we proposed a dual cancelled channel STAP scheme for clutter rejection and slowly moving target detection and localisation in multichannel mobile passive radar. The proposed scheme aimed at reducing the computational complexity, as well as the number of required training data, compared to a conventional full-array solution.

Specifically, the AB-STAP technique was considered, combined with an adjacent-bin post-Doppler strategy, and proved to be a suitable solution for multichannel mobile passive radar. The reduction of computational complexity was obtained both by reducing the number of adaptive DOF in the space-time processing steps and by providing a simple closed-form expression for the target DOA estimation.

Despite its lower computational load, the proposed scheme was shown to yield comparable target detection and DOA estimation performance with respect to the equivalent full array solution. Moreover, it proved to be more robust against adaptivity losses, operating effectively even in the presence of a limited sample support. This plays a fundamental role in a practical passive radar scenario, where the potential non-homogeneity of the bistatic clutter may easily compromise the STAP performance by limiting the number of training data usefully exploitable.

The effectiveness of the proposed scheme was demonstrated against simulated and experimental data from a DVB-T-based multichannel mobile passive radar. The benefits provided were shown to positively contribute to a practical implementation of the system.

## Figures and Tables

**Table 1:** Parameters of the simulated scenario.

Parameters	Values
Receiver altitude	1000 m
Receiver velocity	13 m/s
Carrier frequency	690 MHz
Bandwidth	7.61 MHz
CPI duration	≈0.57 s
Number of channels	4
Element spacing	λ/2

**Table 2:** Parameters of the real and simulated moving targets.

	Delphin	T1	T2	T3	T4
$R_{b}$	6596 m	4700 m	3600 m	6850 m	2400 m
$v_{b}$	−24.5 m/s	−6 m/s	6 m/s	11 m/s	7 m/s
$\phi_{t}$	≈ 37°	37°	157°	109°	70°
$f_{D}$	−31.1 Hz	11.7 Hz	−14.9 Hz	15.9 Hz	26.7 Hz

**Table 3:** Minimum nominal PFA for target detection ($\log_{10}\left(\mathrm{PFA}\right)$ ).

	Full-Array STAP					AB-STAP				
	Delphin	T1	T2	T3	T4	Delphin	T1	T2	T3	T4
K = 24	−7.5	−11.6	−6.7	−10.5	−4.8	−9.6	−9.3	−7.0	−7.8	−5.1
K = 16	−3.1	−6.6	−2.2	−4.6	−3.3	−9.3	−8.6	−8.0	−6.5	−6.6

**Table 4:** Target DOA estimation results.

	Full-Array STAP					AB-STAP				
	Delphin	T1	T2	T3	T4	Delphin	T1	T2	T3	T4
K = 24	34.3° (−2.7°)	36.3° (−0.7°)	158.1° (+1.1°)	108.1° (−0.9°)	69.2° (−0.8°)	35.3° (−1.7°)	37.4° (+0.4°)	157.8° (+0.8°)	109.7° (+0.7°)	69.1° (−0.9°)
K = 16	48.6° (+11.6°)	32.8° (−4.2°)	161.1° (+4.1°)	110.5° (+1.5°)	68.6° (−1.4°)	37.2° (+0.2°)	38.1° (+1.1°)	156.2° (−0.8°)	110.1° (+1.1°)	68.5° (−1.5°)

## Data Availability

Data sharing is not applicable to this article.
